# Behavioural biomechanics: leaf-cutter ant cutting behaviour depends on leaf edge geometry

**DOI:** 10.1098/rspb.2024.2926

**Published:** 2025-04-23

**Authors:** Frederik Püffel, Victor Kang, Mia Yap, Mohammad Shojaeifard, Mattia Bacca, David Labonte

**Affiliations:** ^1^ Department of Bioengineering, Imperial College London, London, UK; ^2^ Department of Mechanical Engineering, University of British Columbia, Vancouver, British Columbia, Canada

**Keywords:** insect–plant interaction, leaf lamina, sheet buckling, cutting forces, behavioural plasticity

## Abstract

Leaf-cutter ants cut fresh leaves to grow a symbiotic fungus as crop. During cutting, one mandible is typically anchored onto the leaf lamina while the other slices through it like a knife. When initiating cuts into the leaf edge, however, foragers sometimes deviate from this behaviour and instead use their mandibles symmetrically, akin to scissors. *In vivo* behavioural assays revealed that the preference for either of the two cutting strategies depended on leaf edge geometry and differed between natural leaf margins that were straight or serrated with notch-like folds: leaf-cutter ants displayed a strong preference for scissor-cutting when leaf edges were straight or had wide notches. This preference, however, reversed in favour of knife-cutting when notches were narrow. To investigate whether this behavioural difference had a mechanical origin, we mimicked knife-cutting in *ex vivo* cutting experiments: for wide notches, all but the sharpest mandibles failed to initiate cuts, or only did so at large forces, caused by substantial leaf buckling and bending. This increased force demand would substantially limit the ability of foragers to cut leaves, and so reduce the colony’s access to food sources. Scissor-cutting may thus be an adaptation to the mechanical difficulties associated with bending and buckling of thin leaves.

## 1. Introduction

Plant-feeding insects play a vital role in most terrestrial ecosystems: they are key pollinators of flowering plants [1], and they substantially accelerate the cycling of carbon, nitrogen and phosphorus [2,3]. However, they also destroy up to one-fifth of our global crop production [4–6], and so cause hundreds of US$ billions in annual economic loss [6,7]. Key to their feeding success is the insects’ ability to overcome the plants’ mechanical defence mechanisms [8,9]: slippery plant surfaces are adhered to via specialized tarsal structures [10,11]; trichomes, small plant-epidermal extensions, are bypassed via elongated rostra [10,12]; and wear-inducing leaf toughness is countered via incorporations of heavy metals, which harden the mandibular cutting edge [13–15]. This evolutionary ‘arms race’ is believed to have substantially driven the unparalleled diversification of both insects and plants [9,16,17].

Insect adaptations for plant-feeding are not only reflected by different genotypes; some developmental, morphological and behavioural adaptations occur plastically in direct response to varying mechanical demands and environmental constraints [18–22]: grass-feeding caterpillars and grasshoppers develop larger heads and mandible closer muscles when reared on ‘harder’ substrates [23,24], allowing faster feeding rates [25]; butterfly larvae that feed on ‘tougher’ or silica-rich leaves grow to smaller sizes, but have longer and relatively heavier mandibles [26]; and cactus bugs grow mouthparts of varying length depending on the wall thickness of the fruits they feed on [27].

An insect herbivore that has adapted particularly well to the challenge of plant foraging are leaf-cutter ants: they consume approximately 15% of the foliar biomass in the Neotropics [28]. Leaf-cutter ants cut small fragments from surrounding vegetation, carry them to a central nest and incorporate them into a fungal garden grown as crop [29–31]. The intricate foraging ecology of leaf-cutter ants is characterized by an extensive division of labour and behavioural plasticity: larger ants cut and carry tougher leaves than smaller morphs from the same colony [29,32,33]; foragers cut smaller fragments when the food source is more attractive [34], closer to the nest [35] or when they face an uphill trail gradient [36] or height obstacles upon return [37]; longer fragments are carried at a steeper neck angle to maintain mechanical stability during walking [38,39]; and foragers modify their leg extension to cut smaller fragments from thicker leaves [40].

The cutting of sheet-like vegetation gives rise to another mechanical challenge that a successful forager must overcome: leaf laminae are only a few hundred micrometres thick [41,42], have weakly constrained margins and thus bend and buckle readily upon the application of even minute forces [43]. How do leaf-cutter ants manage to avoid large deformations that would impede their ability to cut leaves?

**Figure 1.**
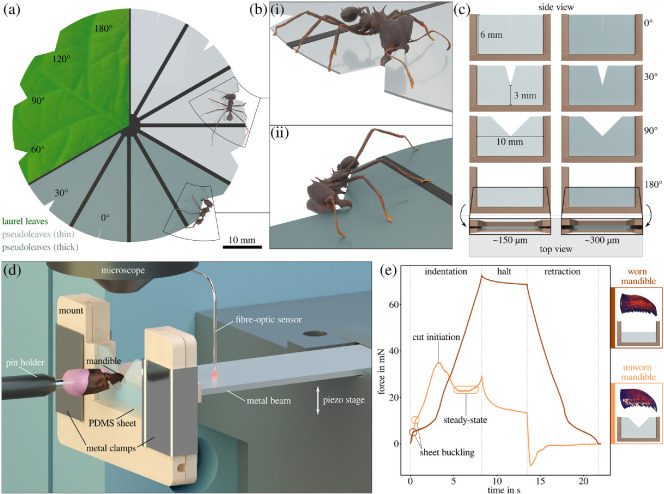
(a) To study the behavioural response of leaf-cutter ants to changes in the mechanical environment during leaf-cutting, we observed how *Atta cephalotes* workers initiated cuts at the edge of thin, leaf-like sheets with varying edge geometry. To this end, we prepared cutting discs (60 mm diameter) from laurel leaves or PDMS pseudoleaves, cut notch angles (0−180°) into the disc edges, and placed the discs inside the colony's foraging arena. (b) From top-down video recordings, two different initiation strategies were identified: (i) knife-cutting, where the long axis of the cutting mandible was perpendicular to the disc plane and (ii) scissor-cutting, where the mandible long axes were approximately in parallel to the disc surface. The three-dimensional reconstructions of the ant models used in this illustration were generated by Fabian Plum using *scAnt* [51]. (c, d) To investigate whether the choice of cutting strategy has a mechanical basis, we performed *ex vivo* cut initiation force measurements using isolated mandibles (see [52] for further details on the sample preparation protocol and experimental set-up). PDMS pseudoleaves of different thickness and notch angles were clamped and mounted onto the free end of a bending beam. The pseudoleaf was then moved against a stationary mandible via a piezo motor stage, so approximating the kinematics during knife-cutting. Upon contact between pseudoleaf and mandible, the beam deflected, as measured with a fibre-optic sensor. The deflection was then converted into a force using a calibration function. (e) Pseudoleaves were either cut (orange line representing an unworn mandible and a approximately 150 µm sheet with 90° notch angle), or merely deformed (mostly out-of-plane; brown line representing a worn mandible and an approximately 150 µm sheet with 180° notch angle); they often bent and buckled out-of-plane, visible by a sudden drop in ‘stiffness’ (circles). When a cut was initiated, the forces typically dropped to an approximately constant level after a peak at initiation (ellipse). When no cut was initiated, the forces instead continued to increase monotonously until the motor stopped; this typically happened when notch angles were large, pseudoleaves thin or mandibles worn. PDMS, polydimethylsiloxane.

Like most biting-chewing insects, leaf-cutter ants possess two mandibles that move predominantly about a single joint axis of rotation [44–47]. The resulting planar kinematic space restricts the ant’s ability to manipulate food items [48], so that the propagation of cuts typically follows a simple stereotyped pattern: the ant anchors one of its mandibles onto the leaf lamina and pushes the other through the lamina akin to a knife slicing through a sheet of paper [49,50]. When initiating cuts into straight leaf edges, however, we observed that ants often deviate from this ‘knife-cutting’ behaviour, and used both mandibles like a pair of scissors instead (figure 1b). After creating a small notch, the ant would then turn to stand on either side of the leaf lamina and continue with knife-cutting. Experimental evidence suggests that introducing a small notch into the sheet edge prior to knife-cutting can mitigate out-of-plane deformations and facilitate cut initiation [50,52–54]. In addition to preliminary scissor-cuts, natural serrations of the leaf margin or previous cutting trajectories lead to variable leaf edge geometries that leaf-cutter ants may encounter during natural foraging. How does this variation, and the associated changes in mechanical constraints, affect the ants’ behaviour at cut initiation? Do leaf-cutter ants perhaps use scissor-cutting to avoid leaf bending and buckling?

To address these questions, we investigated: (i) whether the observed cutting behaviours occurred consistently across different substrates; (ii) how the behavioural preferences changed between serrated instead of straight-edged leaves; and (iii) how the presence and geometry of such serrations affected the biomechanics of cut initiation. By studying the cutting behaviour through a biomechanical lens, we aim to better understand the mechanisms that allow leaf-cutter ants to harvest plants effectively, and, more generally, how physical constraints can drive behavioural adaptations in insect herbivores.

## 2. Material and methods

We initially studied the behaviour of leaf-cutter ants during cut initiation via *in vivo* behavioural assays, where the colony was offered different substrates with varying notch geometries. Informed by observations from these assays, we then designed and performed *ex vivo* cutting force experiments to rationalize the results and to quantify mechanical difficulties at cut initiation.

### (a) Cutting substrate preparation

For behavioural experiments with freely foraging ants, we prepared cutting discs from spotted laurel leaves (*Aucuba japonica*), freshly collected from a single plant near campus (Imperial College London, South Kensington, UK). A circular paper template (60 mm diameter) was used to cut leaf discs from near the leaf base, and the lamina thickness was measured with a digital micrometre (0−25 mm range, ±1 µm, model 293-795-30, Mitutoyo Corporation, Kawasaki, Japan). The average leaf thickness was 390 ± 58 µm (*n* = 52 leaf discs; four replicates per leaf).

During natural foraging, leaf-cutter ants always initiate cuts from the leaf edge [55,56]. The geometry of this edge, however, can vary: for entire leaves with intact margins, the leaf edges are approximately straight; for leaves with serrated margins, at the intersections of previous cuts or when a scissor-cut was made first, the leaf edge has notch-like folds instead. To systematically control for the variation of leaf edge geometry, we cut notches of consistent depth (3 mm), comparable with the mandible length of large *Atta* foragers (approx. 2 mm, [56]) into each of 12 disc sections using a scalpel blade (carbon steel, no. 11, Swann-Morton, Sheffield, UK). Notch angles ranged from a single slit (0° notch angle) to an intact straight edge (180° notch angle—i.e. no notch), with intermediate notch angles of 30°, 60°, 90° and 120°, respectively (figure 1a).

In addition to experiments with laurel leaves, we also used thin sheets of a silicone polymer as cutting substrates (polydimethylsiloxane, PDMS). Synthetic ‘pseudoleaves’ were developed in an effort to minimize uncontrolled covariation present in biological materials: pseudoleaves are homogeneous and can be fabricated with consistent thickness; moreover, PDMS has well-defined mechanical properties, enabling theoretical predictions on cutting force and buckling behaviour. When scented, pseudoleaves are also readily cut by leaf-cutter ant foragers, making them a powerful tool for behavioural assays [35,40].

PDMS sheets were made with a 10 : 1 mixing ratio (base:curing agent; Sylgard 184, Dow Inc., MI, USA), degassed in a vacuum chamber and sandwiched between two silanized glass plates, separated by feeler gauges (150, 200, 300 or 500 µm; Precision Brand, Downers Grove, IL, USA; see [52]). All PDMS samples were cured in an oven at 65°C for 4 h. For *in vivo* behavioural assays, a three-dimensional-printed support structure was embedded into the PDMS prior to curing. This structure consisted of 12 radial spokes, which spanned across a diameter of 60 mm, so creating wheel-like cutting discs akin to leaf laminas supported by veins (figure 1a). We cast cutting discs of two different thicknesses: 160 ± 4 µm (mean ± standard deviation) and 310 ± 5 µm, respectively (*n* = 3 pseudoleaf discs per thickness). Sheet thickness, t, strongly influences its flexural rigidity D ∝ *t*^3^, [43], and thus the resistance against out-of-plane deformation. In analogy to the cutting discs made from laurel, notches were cut into the edges of the cured pseudoleaves. Prior to the behavioural experiments, the pseudoleaf cutting discs were cleaned from any contaminants using 50% ethanol, rinsed with purified water, immersed in honey water for at least 5 min and left to dry at ambient conditions.

For *ex vivo* cut initiation experiments (see below), PDMS sheets of similar thickness were cast (156 ± 3 and 305 ± 3 µm, respectively, *n* = 72 for each), and cut with a scalpel blade to rectangles of 20 × 9 mm (width × height), with central notches of 3 mm length and 0°, 30°, 90° or 180° notch angles. To relate cutting forces to the mechanical properties of PDMS, pure shear tests were performed following the protocol described in Püffel *et al*. [52]. The average critical strain energy release rate, or fracture toughness, was 156 ± 27 J m^−^${,}^{2}$ (*n* = 6, see [54]; the average thickness of the pure shear test samples was 508 ± 7 µm).

### (b) Study animals

A mature *Atta cephalotes* colony was used for all experiments. The colony was kept in a climate chamber at 25°C and 60% relative humidity (FitoClima 12.000 PH, Aralab, Rio de Mouro, Portugal) and fed with bramble, laurel leaves and maize ad libitum.

### (c) Cutting-behavioural assays

*In vivo* cutting assays were conducted in a foraging arena (30 cm × 22 cm), connected to the nest via ≈ 5 m of transparent plastic tubing (24 mm inner diameter). A video camera was positioned above the arena (HQ camera controlled via Raspberry Pi 3A+; Raspberry Pi Foundation, Cambridge, UK), and the camera field of view, focus and exposure times were adjusted prior to each trial. Cutting discs were placed inside the arena on a small three-dimensional printed ‘stool’ to prevent contact between the disc and arena floor. Videos were recorded at 10 fps until cuts had been initiated in each of the 12 disc sections. For each section, the mode of cut initiation was extracted, based on the orientation of the mandible long axis relative to the leaf plane (see below). For all notch angles but 180° (straight edge), only cuts made at the notch centre were considered for further analysis to minimize confounding effects owing to variation in boundary conditions. Experiments were terminated after at least 10 valid cuts per notch angle and substrate were recorded. The median number of valid trials across test conditions was 42, with a minimum and maximum of 10 and 47, respectively.

Per our preliminary observations, ants initiated cuts using one of two strategies (figure 1b): (i) the ant stood on either side of the disc with the long axes of its mandibles approximately perpendicular to the substrate plane. One mandible served as a fixed anchor point towards which the other mandible was then moved to cut the leaf, starting from the leaf edge (knife-cutting, see above and [49,50]); or, (ii) the ant stood on the disc edge with the long axes of its mandibles orientated in parallel to the disc plane, and cuts were made by drawing the mandibles together symmetrically (scissor-cutting). The choice of cut initiation strategy was easily identifiable from the videos.

### (d) Cut initiation force experiments

To probe for a potential mechanical basis for a preference in cut initiation strategy, we performed *ex vivo* cut initiation force measurements with isolated mandibles. The goal was to create a cutting scenario that was mechanically similar to knife-cutting. To this end, we used an experimental set-up initially developed to measure steady-state cutting forces (figure 1d and [52]). In brief, the set-up consisted of a fibre optic displacement sensor that measured the deflection of a bending beam (μDMS-RC32 controlled via DMS Control v. 3.015, Philtec, Annapolis, MD, USA; see the electronic supplementary material for details on sensor calibration and drift correction). Attached to this beam was a polymer mount that held a PDMS pseudoleaf via two metal clamps. Pseudoleaf, beam and sensor were moved upwards by a piezo motor, such that the pseudoleaf was pressed against a stationary mandible, causing the beam to deflect. The mandible was still attached to the head capsule, which was glued onto an insect pin connected to a three-dimensional micromanipulator (see below for details on mandible preparation). The mandible was positioned such that its cutting edge was perpendicular to the sheet, with its distal-most part slightly extending over the sheet edge (akin to knife-cutting, figure 1d). The polymer mount, which held the PDMS pseudoleaf, was U-shaped, with a ‘free’ cutting region of 10 × 6 mm (width × length), large in comparison with the sheet thickness (150–300 µm), and similar to the circumferential distance between neighbouring spokes in the cutting discs (π60mm/12≈16mm). The PDMS sheets were clamped such that the notch was in the centre of the mount, 3 mm away from the lower clamping bar (figure 1c). This distance was chosen to be approximately equal to the maximum mandibular gape of a large *Atta* forager [57]; the clamping bar thus served a similar mechanical function to the anchoring mandible during cut initiation. Experiments that simulate scissor-cutting were attempted, but abandoned owing to substantial technical difficulties associated with small ant heads and brittle apodemes [58].

A total of 18 mandibles were used for these experiments, collected from 18 ant workers that were extracted from a foraging arena connected to the nest by about ≈ 0.5 m of tubing (the arena contained small amounts of fungus, so that some of the collected ants may not have been actively foraging). Ants were sacrificed by freezing, weighed to the nearest 0.1 mg (Explorer Analytical EX124, max. 120 g × 0.1 mg, OHAUS Corporation, Parsippany, NJ, USA; body masses ranged between 17.0 and 37.3 mg), and dissected following the protocol described in Püffel *et al*. [52]. Prior to the cut initiation experiments, individual mandibles were photographed, and steady-state cutting forces measured using PDMS pseudoleaves of an intermediate thickness (207 ± 3 µm). Depending on the extent of mandibular wear, steady-state cutting forces can vary by a factor of about seven among similarly sized *Atta* ants [50,52]. Extracting steady-state in addition to initiation forces thus allowed us to: (i) determine the relative force increase at cut initiation compared with steady-state (see below for details on the calculation); (ii) link the effects of mandibular wear to the probability of pseudoleaf buckling and cut initiation; and (iii) speculate on how mechanics may affect ontogenetic changes in the ants’ cutting behaviour. These separate experiments were necessary, as it was generally not possible to directly measure steady-state cutting forces from the cut initiation experiments; cuts were often not initiated at all, or if they were, the steady-state region was too short to confidently extract an average force (see the electronic supplementary material for more details).

Cut initiation force experiments involved eight measurements per mandible, one for each notch angle and sheet thickness, in randomized order. Individual mandibles were mounted, oriented using a top-down microscope and positioned such that the cutting edge was close to the notch centre (< 50 µm distance, controlled via the motor stage). Subsequently, the force recording was started, and the beam mount was moved against the mandible at a constant rate of 0.3 mm s^−1^, corresponding to the upper end of observed leaf-cutting speeds for larger foragers [32,59]. The motor moved a total distance of 2.5 mm (‘indentation’ phase in figure 1e). To avoid potential damage to the mandible, the motor stopped at a distance of ≈ 0.5 mm to the lower clamping bar (‘halt’ phase). The motor stage then returned to its original position (‘retraction’ phase), and the PDMS sheet was inspected through a microscope to determine if a cut was initiated. Measurements were retaken if: (i) the head capsule came into contact with the mount prior to cut initiation; (ii) the mandible–pin complex moved out of the pin holder; or (iii) the mandible slipped out of the notch, which typically occurred after substantial pseudoleaf buckling.

Where cuts were successfully initiated, we extracted the cut initiation force, $F_{i}$, from the drift-corrected force–time graph. $F_{i}$ was identified by a sharp drop in ‘stiffness’, followed by a short, approximately steady-state region (figure 1e and [60–62]); $F_{i}$ typically corresponded to the maximum measured force during the experiment. The average cutting force was extracted from the steady-state force experiments and rescaled in proportion to sample thickness ($F_{c}^{*}=F_{c}t^{*}/t_{200}$—cutting forces increase in direct proportion to sheet thickness, see [63,64] for experimental verification using *Atta* mandibles). The relative force increase was then calculated as $\Upsilon =F_{i}/F_{c}^{*}$. Normalizing cut initiation forces with steady-state forces allowed us to account for interindividual differences in mandibular wear; a worn mandible requires higher forces to initiate a cut than an unworn mandible, but it also propagates cuts with larger steady-state forces.

### (e) Finite element analysis

The key to cutting success is to avoid large out-of-plane deformations during cut initiation. We systematically tested how notch angle and sheet thickness influence the leaf’s resistance against such deformations by performing finite element analysis: three-dimensional models of both radial disc sections, used for *in vivo* trials, and rectangular sheets, used for *ex vivo* cutting experiments, were constructed in Abaqus/CAE (SIMULIA, Dassault Systèmes SE, Vélizy-Villacoublay, France). Suitable mesh densities were determined via preliminary convergence study; the resulting meshes, for which further refinement was ineffectual, consisted of approximately 136 000 C3D8RH elements (approximately 152 000 nodes) for radial models and approximately 173 000 C3D8RH elements (approximately 194 000 nodes) for rectangular models. Across both geometries, two different sheet thicknesses (150 and 300 µm) and seven notch angles (0°, 30°, 60°, 90°, 120°, 150° and 180°) were simulated, leading to a total of 28 simulations. The material was modelled as linear elastic, and simulations were completed using the static general step. The experimental clamping conditions were mimicked by fixing the node displacements along the left, right and bottom edges in all six degrees of freedom (see figure 1a and c). For each simulation, a uniform out-of-plane displacement (200 µm) was applied to the notch centre, and the resulting reaction forces were extracted. Out-of-plane stiffness was calculated as out-of-plane reaction force divided by the imposed displacement.

In addition to *out-of-plane* deformations, cut initiation also depends on the sheet’s *in-plane* stiffness and the concentration of tensile stresses for crack nucleation [65,66]. We quantified the effects of notch angle and sheet thickness on in-plane stiffness and the resulting tensile stresses by performing a second set of simulations on all rectangular models (14 simulations). The simulation parameters were equal to those used for out-of-plane simulations, except that the displacement at the notch centre (200 µm) was applied in the cutting direction (in-plane). We calculated the in-plane stiffness as in-plane reaction force divided by the imposed displacement and extracted the maximum tensile stress in the pseudoleaf from each simulation.

### (f) Statistical analysis

The relationship between leaf edge geometry and cut initiation strategy was quantified with a binary logistic regression analysis, using notch angle and sheet thickness as independent variables. The logistic function took the form $P(Y)=1/[1+exp(-(b_{0}+b_{1}X_{1}+\cdots +b_{n}X_{n}))]$, where P(Y) is the probability of a knife-like cut initiation, $X_{i}$ represent the predictors (notch angle in degrees and sheet thickness in micrometres) and $b_{i}$ are the respective regression coefficients. Results from *ex vivo* cutting experiments were analysed with repeated measures binary logistic regression analysis, again using notch angle and sheet thickness as fixed effects, and mandible as random effect. Here, the dependent variable encoded whether or not a cut was initiated. To quantify the effects of sheet geometry on the mechanical difficulty to initiate cuts, a linear mixed model was used only on those data where cuts were successfully initiated; the model used the relative force increase, Υ, as dependent variable, notch angle and sheet thickness as fixed effects, and mandible as random effect. Model goodness of fit was assessed via a $\chi^{2}$-test and the Akaike information criterion (AIC). All statistical analyses were conducted in R v. 4.1.2 [67], following Field *et al*. [68]; the R package *lme4* v. 1.1-31 was used for mixed models.

## 3. Results

### (a) Leaf-cutter ants systematically prefer scissor-cuts for sheets with wide notches

**Figure 2.**
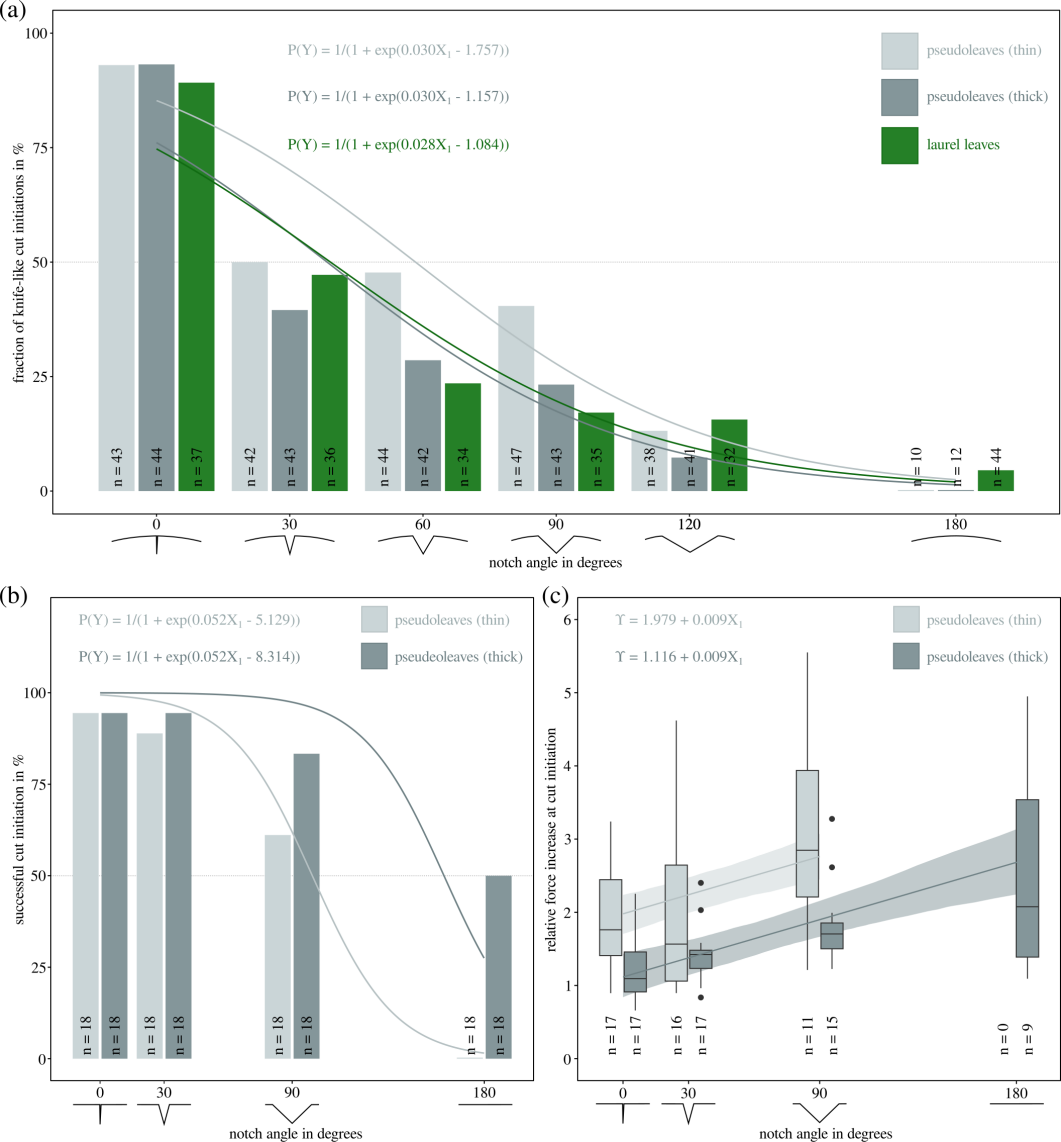
(a) Leaf-cutter ants prefer to initiate cuts with scissor-cutting when notch angles are large, but with knife-cutting when notch angles are small. A binary logistic regression analysis revealed a significant decrease in the probability of knife-cut initiation (P(Y)) with increasing notch angles ($X_{1}$; $b_{1}$= −0.0301 (s.e.: 0.0030) for PDMS pseudoleaves, and $b_{1}$= −0.0277 (s.e.: 0.0041) for laurel leaves). Experiments with PDMS pseudoleaves showed that P(Y) also varied significantly with pseudoleaf thickness, but this effect was small ($X_{2}$; $b_{2}$= −0.0040 (s.e.: 0.0015)). (b) The change in cut initiation preference with edge geometry can be rationalized via results obtained from *ex vivo* cut initiation experiments, designed to imitate knife-cutting. Successfully initiating a cut via knife-cutting was significantly less likely for larger notch angles (P(Y); $b_{1}$= −0.0515 (s.e.: 0.0129)), and significantly more likely for thicker pseudoleaves. Thin pseudoleaves instead often deformed substantially out-of-plane (see the electronic supplementary material, figure S1; $b_{2}$= 0.0214 (s.e.: 0.0067)). (c) Even where cuts were initiated, this involved substantially larger forces, as confirmed by the ratio between cut initiation and steady-state cutting forces, Υ. Υ increased significantly with notch angle (0.0087 (s.e.: 0.0017) per degree angle change), and decreased significantly with sheet thickness (−0.0058 (s.e.: 0.0012) per micrometre thickness change). The increase in cut initiation force may deter, if not physically prevent, leaf-cutter ants from using knife-cutting to initiate cuts into straight leaf edges. This limitation may be particularly restrictive for small ants with worn mandibles, which require a larger fraction of their total bite force capacity to cut the same material [52,57].

*In vivo* cutting behaviour experiments revealed that leaf-cutter ants virtually always used scissor-cutting to initiate cuts into straight leaf edges for both real and pseudoleaves (180${,}^{\circ }$ notch angle, figure 2a). This strong preference steadily declined with decreasing notch angles and ultimately reversed in favour of knife-cutting for 0${,}^{\circ }$ notch angles, where only about 10% of the workers used scissor-cutting to initiate cuts. Accordingly, the logistic regression coefficients for notch angle were significantly negative for both pseudo- and laurel leaves ($b_{1}$ = −0.0301 (s.e.: 0.0030) and $b_{1}$= −0.0277 (s.e.: 0.0041), respectively; see the electronic supplementary material, table S1 for detailed statistics)—the wider the notch angle, the lower the probability of a knife-cut. The notch angles at which both cut initiation strategies occurred with equal probability, P(knife-cut)=P(scissor-cut)=0.5, were 58° (thin pseudoleaves), 38° (thick pseudoleaves) and 39° (laurel leaves), respectively. The preferred strategy also depended significantly on pseudoleaf thickness ($b_{2}$ = −0.0040 (s.e.: 0.0015)): cuts into thick pseudoleaves were slightly less likely to be initiated by a knife-cut than cuts into thin pseudoleaves. However, the associated change in odds per unit increase in thickness, P(knife-cut)/P(scissor-cut), was small (0.996 (confidence interval: 0.993–0.999), where a value of 1 indicates no change).

### (b) Buckling impedes knife-like cut initiation for wide notches

**Figure 3.**
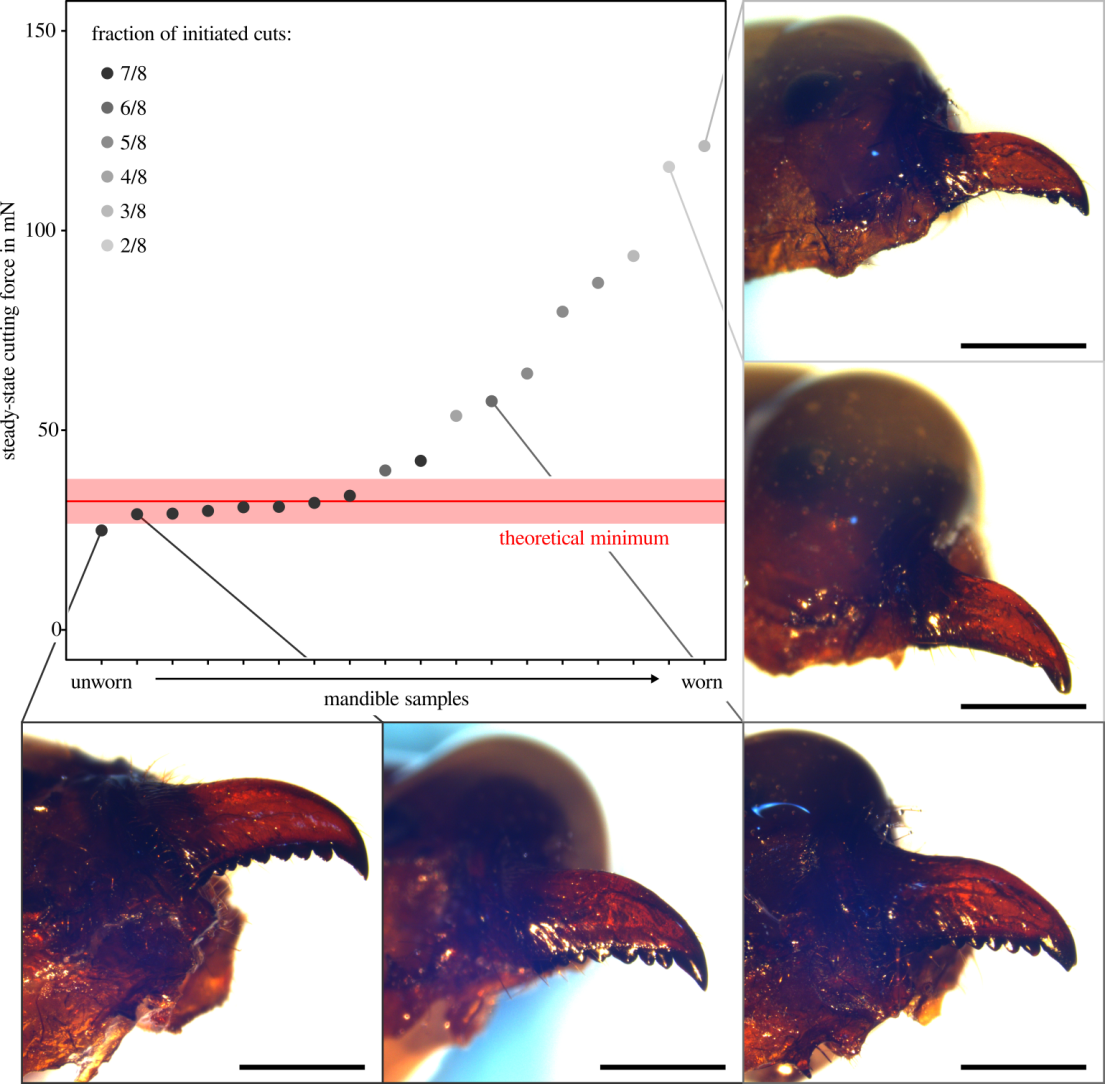
To obtain a proxy for mandible sharpness, steady-state cutting forces were measured for each mandible. Across all mandibles, steady-state forces ranged between 25 and 121 mN. Almost half of the mandibles were cut with approximately the same force, close to a theoretical minimum predicted from the PDMS fracture toughness and sheet thickness (32±6 mN; the shaded area represents the standard deviation calculated as propagated uncertainty following [69]); these mandibles may be considered ‘ideally sharp’ [52]. Larger steady-state forces accordingly occurred for mandibles that were visibly worn; such mandibles also had a significantly lower probability to initiate cuts across all test conditions. Mandibles that required the highest steady-state forces had ‘lost’ almost all their teeth apart from the (distal-most) apical tooth. Scale bars represent a length of 1 mm.

To probe for a potential mechanical underpinning of the behavioural preference for scissor-cuts for large notch angles, we conducted *ex vivo* cutting force experiments that imitated knife-cutting (figure 1d). When pseudoleaves had wider notches, cuts were less likely to be initiated (figure 2b): a unit increase in notch angle was associated with a significant decrease in cut initiation probability ($b_{1}$ = −0.0515 (s.e.: 0.0129)). Sheet thickness, in turn, had a significant positive effect on initiation probability ($b_{2}$ = 0.0214 (s.e.: 0.0067)): the thicker the sheet, the higher the probability of cut initiation. The notch angles at which cuts were initiated half of the time were 100${,}^{\circ }$ and 161${,}^{\circ }$, for thin (approximately 150 µm) and thick (approximately 300 µm) pseudoleaves, respectively. Where cuts failed to initiate, the mandible remained in contact with the notch centre throughout the experiment, but the pseudoleaf deformed substantially out-of-plane instead of being cut (see electronic supplementary material, figure S1 and figure 1e for a typical force profile). Once the motor had returned to its original position, the intact pseudoleaf flattened again, indicating that its deformation had been predominantly elastic. Out-of-plane deformation occurred often even when cuts were successfully initiated. Cut initiation was then typically associated with a sudden reduction in out-of-plane deformation. In cases where some out-of-plane deformation remained visible at the end of the indentation phase, the associated cuts tended to be shorter, as assessed via visual inspection. Shorter cuts and a delayed cut initiation typically occurred for mandibles that required larger steady-state cutting forces (figure 3).

### (c) Knife-like cut initiation requires higher forces when notches are wider

To quantify the mechanical difficulty of cut initiation, we calculated the relative force at initiation compared to steady-state cutting. Steady-state cutting forces ranged from 25 to 121 mN, mirroring previous results ([52]; figure 3). The smallest cutting forces were close to a theoretical minimum estimated from a simple mechanical model that uses PDMS fracture toughness $G_{c}$ and pseudoleaf thickness t [52,63] to estimate $F_{min}=G_{c}t_{200}=32\pm 6$ mN (the standard deviation was calculated as propagated uncertainty following [69]). Mandibles with high steady-state cutting forces had worn and sometimes completely ablated teeth (figure 3), and initiated significantly fewer cuts (Kendall rank correlation: τ=−0.76, *p* < 0.0001, *n* = 18). Across all successful cuts, the relative force increase was 0.7≤Υ≤5.6 (in 13 out of 102 measurements, $F_{i}$ was slightly lower than the steady-state force, $F_{c}^{*}$; in all other cases, Υ>1). The smallest force increase was observed for thick pseudoleaves with narrow notches, where Υ≈1—the force at cut initiation was about equal to the steady-state cutting force. Υ increased significantly with notch angle (0.0087 (s.e.: 0.0017) per degree change in angle), approaching 3 for thick pseudoleaves with straight edges (figure 2c): the wider the notch, the more adverse the effect of sheet deformation on cut initiation force. Υ was also higher for thinner pseudoleaves: cut initiation forces were approximately double the steady-state cutting force for thin pseudoleaves with 0${,}^{\circ }$ notch angles (Υ≈2), and increased to almost three times compared with the steady-state cutting force for 90${,}^{\circ }$ notch angles (slope: −0.0058 (s.e.: 0.0012) per micrometre change in thickness). Υ could not be evaluated for thin pseudoleaves with straight edges because none of the 18 tested mandibles successfully initiated a cut. Both notch angle and sheet thickness thus had a significant effect on Υ; accordingly, the variation of Υ was best described by a linear mixed model with both notch angle and sheet thickness as fixed effects, $f(X_{1},X_{2})$, compared with a model with notch angle as a single predictor, $f(X_{1})$, or an intercept-only model ($f(X_{1})$ versus ‘intercept’: $\chi_{1}^{2}$ = 13.37, *p* < 0.001, AIC (290.0 versus 301.4); $f(X_{1},X_{2})$ versus $f(X_{1})$: $\chi_{1}^{2}$ = 20.81, *p* < 0.0001, AIC (271.2 versus 290.0)).

### (d) Out-of-plane and in-plane stiffness decrease substantially with increasing notch angle

**Figure 4.**
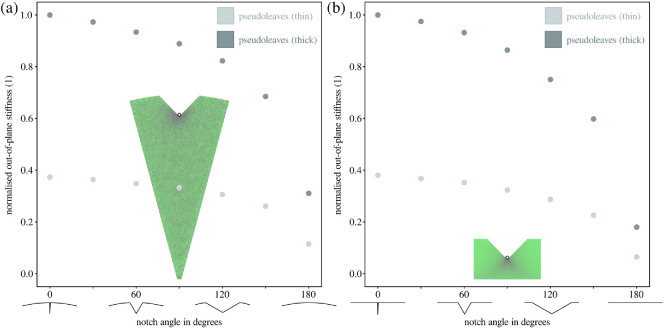
To further explore the mechanical underpinning of the observed differences in cutting strategy and cut initiation forces, we performed finite element analysis. Three-dimensional models of both radial disc sections—(a) *in vivo* behavioural assays—and rectangular sheets—(*b*) *ex vivo* cutting experiments—were subjected to a fixed out-of-plane displacement at the notch centres. The resulting reaction force per unit displacement, the out-of-plane stiffness, was maximum for thick sheets with narrow notches and monotonously decreased with increasing notch angle. For both radial and rectangular geometries, the values for out-of-plane stiffness were normalized with their respective maxima. In general, thick sheets had a larger out-of-plane stiffness than thin sheets (≈ 2.7 times). Across both geometries and sheet thicknesses, the out-of-plane stiffness decreased by a factor 3−6 between 0${,}^{\circ }$ and 180${,}^{\circ }$ notch angles, suggesting that sheets with narrow notches have a substantially higher resistance against bending and buckling. This increased resistance against out-of-plane deformation, in conjunction with higher maximum tensile stresses (see electronic supplementary material, figure S2b), probably facilitates knife-like cut initiation, in line with both behavioural observations—knife-cuts were preferred for sheets with sharp notches (figure 2a), and cutting experiments—the force at cut initiation was lowest for 0${,}^{\circ }$ notch angles (figure 2c).

The effects of notch angle on the sheet’s resistance against out-of-plane and in-plane deformation were further investigated with finite element analysis. Out-of-plane stiffness varied strongly between thin and thick pseudoleaves across both geometries: for radial disc sections, it was 2.68 ± 0.02 times larger for thicker sheets, almost identical to the results obtained for rectangular sheets (2.66 ± 0.06; see figure 4). The out-of-plane stiffness was also substantially affected by notch angle: across both geometries and sheet thicknesses, out-of-plane stiffness was maximum for sheets with sharp notches, and monotonously decreased with increasing notch angle to less than one-third for straight leaf edges (≈ 0.31 and ≈ 0.17 for radial and rectangular geometries, respectively). The largest drop in stiffness between two consecutive angle increments occurred consistently between 150° and 180°, with a drop of ≈ 55% and ≈ 71% for radial and rectangular geometries, respectively.

In analogy to these results, sheet thickness and notch angle also affected the resistance against in-plane deformation. In-plane stiffness was consistently larger for thicker sheets (1.99 ± 0.03; see the electronic supplementary material, figure S2a), and monotonously decreased from its maximum at 0${,}^{\circ }$ notch angle to approximately half of that for straight edges. As a result of this increased in-plane stiffness, the maximum tensile stresses were substantially larger for sharp notches than for wide notches at equivalent indentation depths (factor of ≈ 3 between 0° and 180° notch angles, see electronic supplementary material, figure S2b).

## 4. Discussion

The mechanical demands of plant foraging have shaped the evolution of mouthpart morphology and the feeding behaviour across the herbivorous Insecta [10,46,48,70,71]. Some herbivores, such as leaf-cutter ants, display a diverse set of adaptations to meet these demands effectively: leaf-cutter ant colonies deploy a polymorphous workforce to forage on plant materials of variable properties, where larger workers cut and carry tougher leaves [29,32,33]; the musculoskeletal bite apparatus is specialized to produce exceptionally large weight-specific bite forces [57,72–74], and to cut leaves with lower forces than pristine scalpel blades [50,52]; leaf-cutter ant foragers favour pre-cut leaf fragments over intact leaves which would require more cutting [75]; and they adjust their gaits and neck positions to the size and shape of the carried fragments [39].

In this study, we show that leaf-cutter ants also adjust their cutting strategy to overcome a mechanical difficulty specific to thin-sheet cutting: workers tend to prefer scissor- over knife-cuts when the risk of out-of-plane deformation is high. This strong behavioural preference can be rationalized with results from *ex vivo* cutting experiments that mirrored knife-cutting: an increase in notch angle was associated with a decrease in initiation probability and a substantial increase in the cut initiation force. In the following sections, we discuss the adverse consequences of increased cut initiation forces for the colony’s ability to access food sources and explore the potential mechanical basis for behavioural plasticity during cut initiation.

### (a) Lower cut initiation forces may increase the range of accessible food sources

Leaf-cutter ants can only cut leaves if they can generate sufficiently large bite force. The required bite force generally depends on the material properties of the leaf and the geometry of the cutting mandible. Based on *in vivo* bite force measurements [57], combined with leaf-mechanical data [41], we previously estimated the typical force a leaf-cutter ant forager may have to generate to cut the median tropical leaf as about 82 mN [57]. The force at cut initiation for a 0° notch was $\Upsilon =2.883+0.009(0^{\circ })-0.006(\text{median leaf thickness}$ = 210 µm) ≈ 1.67 larger than the typical steady-state force measured for the same material. Extrapolating from this data, the minimum ant body mass necessary to initiate a cut via knife-cutting may be estimated from the bite force data as about 6 mg (for more details, see the electronic supplementary material). For a straight leaf edge (180${,}^{\circ }$ notch angle), this force would increase instead by a factor Υ≈3.24 and the minimum body mass to about 12 mg—twice as heavy as for a narrow notch, and almost four times as heavy as required for steady-state cutting forces. If these differences appear small, consider the reduction in accessible plant material: a worker ant with a body mass of 6 mg can generate bite forces sufficient for the steady-state cutting of 78% of tropical leaves, but can only initiate knife-cuts into a meagre 16%—and no steady-state cutting is possible without initiating a cut. The large cut initiation forces associated with out-of-plane sheet deformation may thus effectively prevent small ants from cutting leaves with straight edges altogether. Larger ants are probably less affected by this force increase: owing to an increased bite force capacity, a 30 mg ant could still initiate knife-cuts into 88% of tropical leaves with straight edges, compared with 98% for leaves with narrow notches. However, the increased forces and associated energetic costs may still drive large ants to use scissor-cutting instead; we caution against definitive conclusions on the energetic benefits of scissor-cutting, which require either *in vivo* metabolic rate measurements during cut initiation, or experiments with a force measurement setup that imitates scissor-cutting.

An ant’s ability to initiate knife-cuts is affected not only by its body size but also by the wear state of its mandibles. Sharp mandibles, with low steady-state cutting forces, successfully initiated cuts across all test conditions, except for thin sheets with straight edges. By contrast, worn mandibles with high steady-state forces only initiated cuts when notches were narrow ($\leq 30^{\circ }$, figures 2b and 3). Knife-like cut initiation is thus particularly difficult for foragers with worn mandibles, and this difficulty may drive older ants to avoid this strategy altogether [50,76,77]. Across active foragers, the distribution of mandibular wear states may be partially reflected by the statistical distribution of behavioural responses observed during the cutting assays (figure 2a): ants with particularly worn mandibles may comprise the small fraction of foragers that still deployed scissor-cutting even when notch angles were small. Conversely, the few ants that used knife-cutting for pseudoleaves with wide notches may have possessed almost pristine mandibles, so that cut initiation occurred at loads too small to cause excessive out-of-plane deformations. In addition to variation in mandibular wear, the ants’ behavioural preference may also be influenced by size-related differences in bite force (see above), individual preferences and local environmental conditions: narrow notches may render the ant’s access to the notch centre difficult, so that knife-cuts are simply more practical. The ability to anchor the legs onto the sheet edge, as well as interactions with other ants may have further contributed to a ‘softer’ transition between the two initiation strategies.

### (b) Biomechanics of cut initiation—what are the advantages of scissor-cuts?

The results from *ex vivo* cutting experiments provide strong arguments *against* the use of knife-cutting when leaf edges are straight or have wide notches: large out-of-plane deformations lead to increased cut initiation forces or even prevent cut initiation altogether. However, what are the arguments *for* using scissor-cutting instead?

To approach this question from a mechanical angle, we first note that knife- and scissor-cutting are not mechanically equivalent—they differ in their respective modes of fracture (mode I versus mode III), blade orientation relative to the sheet plane, and ‘slice-to-push’ ratios [66,70,78–80]. However, both cutting modes involve material fracture and thus the creation of new surface area. Each unit area, dA of new surface demands a minimum energy investment, $dU_{A}=G_{c}dA$, which must be supplied by the cutting mandible; additional energy sinks may emerge from friction and non-recoverable elastic strain energy [63,78]. Although simple, such virtual work arguments have been successfully used to demonstrate that pristine leaf-cutter ant mandibles are close to optimally ‘sharp’, i.e. steady-state forces during knife-cutting of PDMS pseudoleaves were close to a theoretical minimum, $F_{c}\approx F_{min}=G_{c}t$ [52].

During scissor-cutting, additional energetic costs may arise from friction between the overlapping blades: industrial scissors are often spring-loaded to enable ‘clean’ cuts of thin sheets [80–82], and leaf-cutter ant mandibles contact and elastically pitch out-of-plane when they overlap [47]. Even if this frictional contribution was negligible, the energy required to scissor-cut would still be bound from below by $G_{c}dA$, the minimum achieved by almost half of the mandibles during knife-cutting (figure 3). For sharp mandibles, scissor-cutting may thus require at least as much energy per cut area as knife-cutting during cut propagation. However, what about cut initiation?

Our experiments demonstrated that knife-cuts can be difficult, if not impossible to initiate, because thin sheets have low out-of-plane and in-plane stiffness, and thus easily bend and buckle before nucleating cracks; buckling loads generally increase with the flexural rigidity, $D\propto t^{3}$ [43]. Leaf-cutter ants may thus prefer scissor-cuts, because the mandibles then locally ‘clamp’ the sheet, so avoiding this problem altogether. Even where knife-cuts remain possible, sheet bending will incur additional elastic energy penalties, increase the cut initiation force required to meet the stress demands for crack nucleation, and thus enlarge the total energetic costs associated with cut initiation. These adverse effects can be mitigated by introducing notches into the sheet edge (either artificially or via a preceding scissor-cut [50–53]); notches can increase the out-of-plane stiffness by almost a factor of six and the in-plane stiffness by a factor of two (see figure 4b and the electronic supplementary material, figure S2).

The results from *ex vivo* cut initiation experiments and *in silico* numerical simulations demonstrate that the mechanics of cut initiation are affected not only by the presence of a notch but also by its angle. The narrower the angle, the larger the out-of-plane and in-plane stiffness (see figure 4 and the electronic supplementary material, figure S2), which increases the probability of cut initiation and minimizes the relative force increase (figure 2b,c). The notch angles at which 50% of cuts were initiated (P(Y)=0.5) were between $90^{\circ }$ and $180^{\circ }$. This transition occurred at larger angles than the transition between *in vivo* cutting strategies (P(Y)=0.5 at $30-60^{\circ }$, figure 2a), indicating that some ants may have preferred scissor-cuts over knife-cuts, even when knife-cuts could have been initiated. This sustained preference for scissor-cuts may thus be driven by the need to initiate cuts with low forces more so than by the ability to initiate cuts at all.

## 5. Conclusion and outlook

Leaf-cutter ants use different cutting strategies to forage on thin leaves. Cuts into leaves with wide notches are typically initiated using scissor-cutting, before the ant positions itself on either side of the leaf lamina and propagates the cut via knife-cutting [49,50]. Leaves with narrow notches, in turn, are knife-cut right away. This behavioural difference can be rationalized by considering the mechanics of thin-sheet cutting: knife-cutting thin sheets with wide notches requires high initiation forces—if cuts are initiated at all. The effects of an increased initiation force can be substantial: smaller ants would no longer be able to initiate cuts into two-thirds of the plant species they are otherwise able to cut, and this substantial negative effect provides a plausible explanation for the strong preference for scissor-cuts. We thus posit that scissor-cutting may have partially evolved as a strategy to avoid large out-of-plane deformations when initiating cuts into thin leaf laminae and flower petals. There are other potential behaviours that could reduce the risk of buckling, such as leaf edge support using the limbs, or a variation in mandible kinematics during cutting. These behaviours may be explored in further work.

## Data Availability

All data are provided in the electronic supplementary material [83].
